# Immediate improvements in post-stroke gait biomechanics are induced with both real-time limb position and propulsive force biofeedback

**DOI:** 10.1186/s12984-023-01154-3

**Published:** 2023-03-31

**Authors:** Vincent Santucci, Zahin Alam, Justin Liu, Jacob Spencer, Alec Faust, Aijalon Cobb, Joshua Konantz, Steven Eicholtz, Steven Wolf, Trisha M. Kesar

**Affiliations:** 1 Division of Physical Therapy, Department of Rehabilitation Medicine, Emory University, Atlanta, GA USA; 2 Center for Visual and Neurocognitive Rehabilitation, VA Medical Center, Atlanta, GA USA; 3 Emory Rehabilitation Hospital, 1441 Clifton Rd NE, Atlanta, GA 30322 USA

**Keywords:** Stroke gait rehabilitation, Trailing limb angle, Pushoff, Feedback

## Abstract

**Background:**

Paretic propulsion [measured as anteriorly-directed ground reaction forces (AGRF)] and trailing limb angle (TLA) show robust inter-relationships, and represent two key modifiable post-stroke gait variables that have biomechanical and clinical relevance. Our recent work demonstrated that real-time biofeedback is a feasible paradigm for modulating AGRF and TLA in able-bodied participants. However, the effects of TLA biofeedback on gait biomechanics of post-stroke individuals are poorly understood. Thus, our objective was to investigate the effects of unilateral, real-time, audiovisual TLA versus AGRF biofeedback on gait biomechanics in post-stroke individuals.

**Methods:**

Nine post-stroke individuals (6 males, age 63 ± 9.8 years, 44.9 months post-stroke) participated in a single session of gait analysis comprised of three types of walking trials: no biofeedback, AGRF biofeedback, and TLA biofeedback. Biofeedback unilaterally targeted deficits on the paretic limb. Dependent variables included peak AGRF, TLA, and ankle plantarflexor moment. One-way repeated measures ANOVA with Bonferroni-corrected post-hoc comparisons were conducted to detect the effect of biofeedback on gait biomechanics variables.

**Results:**

Compared to no-biofeedback, both AGRF and TLA biofeedback induced unilateral increases in paretic AGRF. TLA biofeedback induced significantly larger increases in paretic TLA than AGRF biofeedback. AGRF biofeedback increased ankle moment, and both feedback conditions increased non-paretic step length. Both types of biofeedback specifically targeted the paretic limb without inducing changes in the non-paretic limb.

**Conclusions:**

By showing comparable increases in paretic limb gait biomechanics in response to both TLA and AGRF biofeedback, our novel findings provide the rationale and feasibility of paretic TLA as a gait biofeedback target for post-stroke individuals. Additionally, our results provide preliminary insights into divergent biomechanical mechanisms underlying improvements in post-stroke gait induced by these two biofeedback targets. We lay the groundwork for future investigations incorporating greater dosages and longer-term therapeutic effects of TLA biofeedback as a stroke gait rehabilitation strategy.

*Trial registration* NCT03466372

## Introduction

Hemiparesis following stroke causes unilateral deficits in gait kinematics and kinetics, contributing to slowed gait speed, gait asymmetries, and increased fall risk [1–3]. While increasing gait speed is a major goal of stroke rehabilitation [4, 5], improvements in speed can be achieved either through restoration of paretic limb function or compensatory strategies [6]. Measurement of kinematic and kinetic gait biomechanics variables can parse out restoration versus compensation as sources of gait recovery or training-induced improvements [7]. Reduced paretic propulsion, measured as the anterior component of the ground reaction force (AGRF) generated during late stance, is an important biomechanical deficit closely associated with gait speed and walking function post-stroke [8–11]. Importantly, individuals post-stroke demonstrate a paretic propulsive reserve [12] that can be exploited using gait training interventions [13, 14], with improvements in propulsion correlating to improvements in gait speed [8]. Thus, propulsion has emerged as a key modifiable post-stroke gait variable that is biomechanically and clinically relevant.

Previous studies have demonstrated two major biomechanical gait variables that contribute to overall propulsion, ankle plantarflexor moment, and trailing limb angle [15]. Ankle plantarflexors generate most of the force required to facilitate a smooth stance-to-swing transition [16]. Trailing limb angle (TLA), a measure of the overall limb angle or position with respect to the center of mass, places the leg in a better orientation to direct ground reaction forces more anteriorly [17]. Individuals post-stroke demonstrate deficits in both paretic plantarflexor moment and TLA [15], yet increases in paretic propulsion appear to originate mainly from improvements in paretic TLA [12, 18]. Post-stroke AGRF and TLA show robust inter-relationships, indicating that TLA can be used as a surrogate for paretic AGRF measurements [19]. Taken together, these studies suggest that targeting post-stroke TLA deficits may be a feasible and effective way of improving paretic propulsion.

Real-time biofeedback has emerged as a promising post-stroke gait training strategy that can target specific gait deficits on the paretic limb [20–23]. Previously, unilateral biofeedback targeting paretic propulsion was shown to induce significant increases in paretic limb propulsion without concomitant compensatory changes in the non-paretic limb [20]. However, translation of propulsion biofeedback from laboratory to clinic remains difficult because laboratory-based instrumented walkways or treadmills needed to measure AGRF may not be clinically accessible. The use of portable and wearable AGRF sensors for gait assessment is under study and not yet clinically available [24]. Moreover, estimation of propulsion through observational gait analysis is challenging even for movement experts with considerable clinical experience [25]. In contrast, measurements of TLA do not require the use of force platforms, and can be more easily subjectively estimated by clinicians based on the relationship of the forefoot to the pelvis or greater trochanter during observational gait analysis [19]. Recently, we demonstrated that able-bodied individuals are able to modulate TLA and AGRF unilaterally in response to real-time unilateral TLA biofeedback [26]. Thus, TLA biofeedback holds promise as a clinically applicable intervention that could preferentially increase paretic AGRF and reduce post-stroke propulsion deficits. While AGRF and TLA have been studied together as outcome variables in previous research, to our knowledge, biofeedback for these 2 biomechanical targets has not been directly compared in people post-stroke. Thus, an initial assessment of the feasibility and immediate biomechanical effects of TLA biofeedback on post-stroke individuals is needed.

Here, we studied the effects of TLA biofeedback on post-stroke gait biomechanics. Moreover, to assess its use as a suitable and clinically applicable alternative to AGRF biofeedback, we compared the immediate biomechanical effects of TLA biofeedback to AGRF biofeedback. We hypothesized that a biofeedback paradigm targeting paretic TLA would elicit favorable improvements in paretic propulsion and other post-stroke gait biomechanics impairments that are comparable in magnitude to AGRF biofeedback.

### Methods

Nine individuals with chronic post-stroke lower extremity hemiparesis (6 males, age 63 ± 9.8 years, 44.9 months post-stroke) participated in this study (Table 1). Inclusion criteria were: > 6 months post-stroke, ability to walk on a treadmill continuously for 1 min, and ability to effectively communicate with investigators. Exclusion criteria included neurologic diagnosis other than stroke, orthopedic conditions limiting walking, and cerebellar dysfunction. All participants provided informed consent and the study was approved by the institutional Human Subjects Review Board.

**Table 1:** Participant demographics and clinical characteristics

Participant	Gender	Age (years)	Time post-stroke (months)	Affected side	Berg balance scale score (Max 56)	Lower extremity Fugl-Meyer score (Max 34)	Treadmill self-selected walking speed (m/s)
1	Male	74	106	Right	50	22	0.35
2	Female	67	65	Left	48	20	0.6
3	Female	74	24	Left	54	26	0.45
4	Male	53	7	Left	46	15	0.25
5	Male	58	6	Left	56	26	0.9
6	Female	58	64	Left	34	18	0.35
7	Male	75	8	Left	43	22	0.4
8	Male	59	46	Left	42	23	0.37
9	Male	49	78	Right	45	17	0.3
Avg		63	44.9		46.4	21	0.44
Std dev		9.8	36		6.6	3.8	0.2

### Marker set-up

Reflective markers were attached to the trunk, pelvis, and bilateral thigh, shank, and foot segments [27]. Marker position data were captured using a 7-camera motion capture system (Vicon Inc., Colorado, USA). Participants walked on a dual-belt treadmill embedded with force platforms to collect ground reaction force data from each limb (Bertec Corporation, Ohio, USA). All participants were provided an overhead harness without bodyweight support and a front handrail for safety. Participants were instructed to keep a light fingertip touch on the handrail during all walking trials. If participants modified their hand grip or exhibited excessive trunk lean during a walking trial, the trial was stopped and performed again.

### Experimental protocol

At the beginning of the session, the self-selected walking speed of participants was determined by increasing the treadmill speed by 0.1 m/s increments until participants reported their comfortable walking speed. All subsequent walking trials were performed at each participant’s self-selected walking speed (Table 1). Next, participants completed three 60-s walking trials in the following order: no biofeedback exposure (baseline), AGRF biofeedback, and TLA biofeedback. During the baseline trial, participants were instructed to walk naturally. Baseline peak AGRF and peak TLA data averaged across all strides were extracted from the baseline trial. Biofeedback targets for AGRF biofeedback and TLA biofeedback were calculated as 25% greater than baseline AGRF and TLA [20, 26, 28], For two participants, paretic limb AGRFs were too low for a target set at 25% above baseline; thus, targets were set to either 25% or 50% of inter-limb AGRF deficit. The target value was confirmed to be at an appropriate challenge level for each participant based on success rate, clinical judgement, and participant feedback during an initial familiarization trial. Thus, the feedback target value was individualized so as to provide biofeedback that could shape short-term gait changes customized according to each individual’s neuromechanical gait deficits. Prior to each biofeedback trial, participants were given scripted instructions regarding the biofeedback interface, the limb that would receive biofeedback (paretic limb), and the objective of the gait trial. The methods used were similar to those for our previous able-bodied study [26]. Participants were provided with a 2-min standing break after each walking trial.

### Biofeedback methodology

For walking trials with biofeedback, all participants were provided with real-time audiovisual biofeedback displaying either real-time AGRF or TLA information for the hemiparetic limb [26]. For AGRF biofeedback, the visual display consisted of a horizontal line with a cursor (X) that represented real-time magnitude of paretic limb AGRF [26]. Increasing paretic AGRF moved the cursor towards the AGRF target range, represented by a green line with a 5-Newton error-tolerance range centered around the target. Audio biofeedback consisted of an audible beep that played when the cursor entered the AGRF target range. For TLA biofeedback, the visual display consisted of a horizontal line with a cursor that represented the ongoing paretic limb TLA [26]. The target TLA biofeedback target consisted of a green line with a 2-degree error-tolerance range centered around the target. Audio biofeedback comprised an audible beep indicating success when the cursor entered the TLA target range.

### Dependent variables

Primary dependent variables consisted of peak paretic AGRF, TLA, and ankle plantarflexor moment during late stance. Secondary variables included peak non-paretic AGRF, TLA, and ankle plantarflexor moment during late stance. Peak AGRF was calculated as the peak anterior force generation between contralateral heel strike and ipsilateral toe-off averaged across all strides. Peak TLA was calculated as the maximum angle between the greater trochanter and fifth metatarsal head marker averaged across all strides. Peak ankle plantarflexor moment was calculated as the peak plantarflexor moment at terminal stance averaged across all strides. Additionally, percent paretic propulsion was calculated as the peak paretic AGRF divided by the sum of the peak AGRFs for the paretic and non-paretic limbs. A value of 50% paretic propulsion indicates equal contribution to propulsion from the paretic and non-paretic limbs. Inter-limb TLA asymmetry and peak ankle moment asymmetry were calculated as the difference between peak non-paretic and paretic limbs. A positive value indicates a larger non-paretic vs. paretic value, zero indicates symmetry between both limbs, and a negative value indicates a larger paretic vs. non-paretic value. Finally, paretic and non-paretic step lengths were calculated as the antero-posterior distance between the bottom heel markers of the trailing and leading leg, with the leading leg used as reference. Inter-limb differences in step length were calculated as paretic step length ratio [paretic/(paretic + nonparetic step length)].

### Statistical analyses

One-way repeated measures ANOVAs were performed to evaluate the effect of biofeedback on each dependent variable. Post-hoc Bonferroni-corrected pairwise comparisons were performed if a significant main effect of biofeedback was demonstrated. All statistical analyses were performed in IBM SPSS Statistics (IBM, New York, USA). Alpha level was set at 0.05 for all statistical tests.

## Results

### Paretic and non-paretic limb peak AGRF

**Fig. 1:**
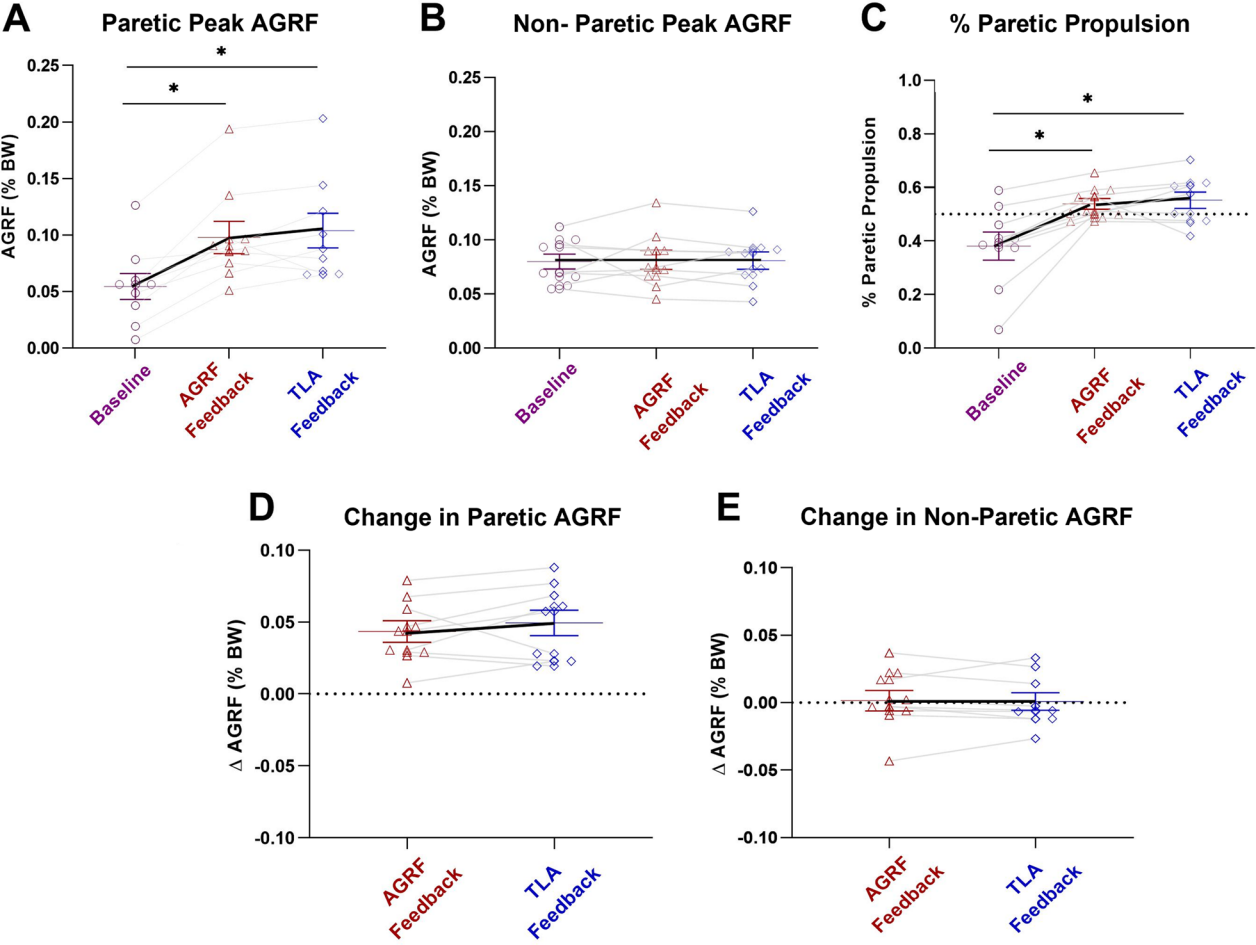
Peak AGRF (average ± standard error) for the **A** paretic limb, **B** non-paretic limb, **C** % paretic propulsion during baseline (no-biofeedback), AGRF biofeedback, and TLA biofeedback gait trials, as well as **D** change in paretic and **E** change in non-paretic AGRF with biofeedback. The one-way repeated measures ANOVA demonstrated a significant main effect of biofeedback condition on paretic limb peak AGRF (p < 0.001). The symbol * indicates pairwise comparisons that showed significant differences between conditions. Both AGRF biofeedback (p = 0.001) and TLA biofeedback (p = 0.001) induced significant increase in paretic limb peak AGRF compared to baseline. There was a significant main effect of biofeedback on % propulsion contributed by the paretic limb (p < 0.001), with both AGRF biofeedback (p = 0.01) and TLA biofeedback (p = 0.015) inducing significant increases in % propulsion compared to baseline

The one-way repeated measures ANOVA demonstrated a significant main effect of biofeedback on paretic limb peak AGRF (p < 0.001, F = 25.554). Bonferroni-corrected pairwise comparisons revealed a significant increase in paretic limb peak AGRF during the AGRF biofeedback trial compared to baseline (p = 0.001) (Fig. 1A). Additionally, pairwise comparisons revealed a significant increase in paretic limb peak AGRF during the TLA biofeedback trial compared to baseline (p = 0.001). No significant differences in paretic limb peak AGRF were observed between the AGRF and TLA biofeedback walking trials (p = 1.000).

The one-way repeated measures ANOVA did not show a significant main effect of biofeedback on non-paretic limb peak AGRF (p = 0.967, F = 0.033) (Fig. 1B).

### Percentage of propulsion generated by the paretic limb

The one-way repeated measures ANOVA demonstrated a significant main effect of biofeedback on percentage of propulsion by the paretic limb (p < 0.001, F = 13.591). Bonferroni-corrected pairwise comparisons revealed a significant increase in percentage of propulsion by the paretic limb during the AGRF biofeedback trial compared to baseline (p = 0.01) (Fig. 1C). Additionally, pairwise comparisons revealed a significant increase in percentage of propulsion by paretic limb AGRF during the TLA biofeedback trial compared to baseline (p = 0.015). No significant differences in percentage of propulsion by paretic limb were observed between the AGRF and TLA biofeedback walking trials (p = 1.000).

### Paretic and non-paretic limb peak TLA

**Fig. 2:**
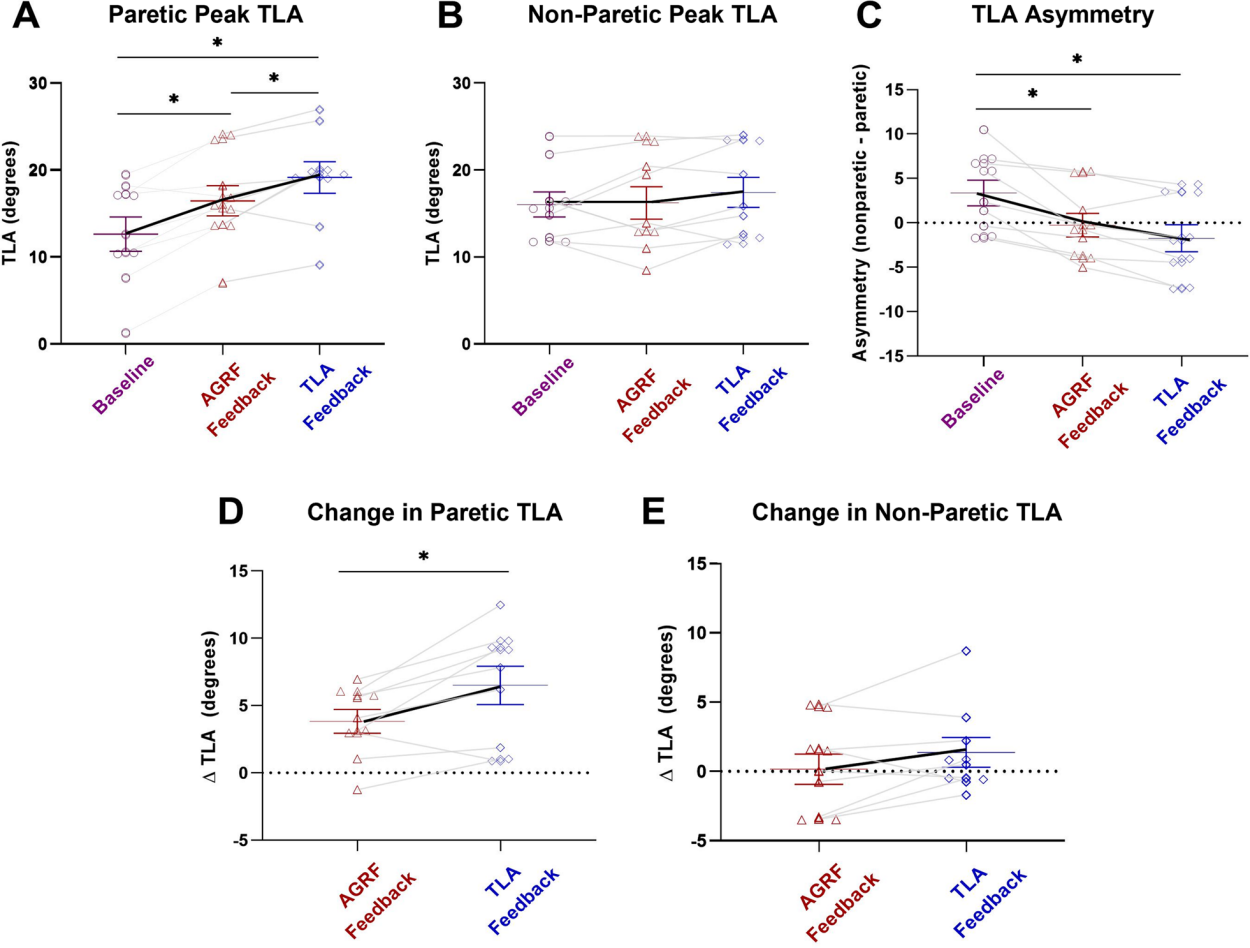
Peak TLA (average ± standard error) for the **A** paretic limb, **B** non-paretic limb, and **C** TLA asymmetry during baseline (no-biofeedback), AGRF biofeedback, and TLA biofeedback gait trials, as well as **D** change in paretic TLA, and **E** change in non-paretic TLA with biofeedback. The one-way repeated measures ANOVA demonstrated a significant main effect of biofeedback on paretic limb peak TLA (p < 0.001). The symbol * indicates pairwise comparisons that showed significant differences between conditions. Both AGRF biofeedback (p = 0.008) and TLA biofeedback (p = 0.006) induced significant increase in paretic limb peak TLA compared to baseline, with TLA biofeedback significantly increasing (p = 0.045) paretic peak TLA compared to AGRF. Both AGRF biofeedback (p = 0.017) and TLA biofeedback (p = 0.002) significantly improved TLA asymmetry compared to baseline, with TLA biofeedback significantly improving TLA asymmetry compared to AGRF biofeedback

The one-way repeated measures ANOVA demonstrated a significant main effect of biofeedback on paretic limb peak TLA (p < 0.001, F = 17.974). Pairwise comparisons showed a significant increase in paretic limb peak TLA during the AGRF biofeedback trial compared to baseline (p = 0.008) (Fig. 2A). Additionally, pairwise comparisons showed a significant increase in paretic limb peak TLA during the TLA biofeedback trial compared to baseline (p = 0.006). A significant increase in paretic limb peak TLA was observed during the TLA biofeedback trial compared to the AGRF biofeedback trial (p = 0.045).

The one-way repeated measures ANOVA did not show a significant main effect of biofeedback on non-paretic limb peak AGRF (p = 0.328, F = 1.195) (Fig. 2B).

### Interlimb TLA asymmetry

The one-way repeated measures ANOVA demonstrated a significant main effect of biofeedback on interlimb TLA asymmetry (p < 0.001, F = 18.520). Bonferroni-corrected pairwise comparisons revealed a significant increase in interlimb asymmetry of TLA during the AGRF biofeedback trial compared to baseline (p = 0.017) (Fig. 2C). Additionally, pairwise comparisons revealed a significant increase in interlimb asymmetry of TLA during the TLA biofeedback trial compared to baseline (p = 0.002). No significant differences in interlimb asymmetry of TLA were observed between the AGRF and TLA biofeedback walking trials (p = 0.114).

### Paretic limb peak ankle moment

**Fig. 3:**
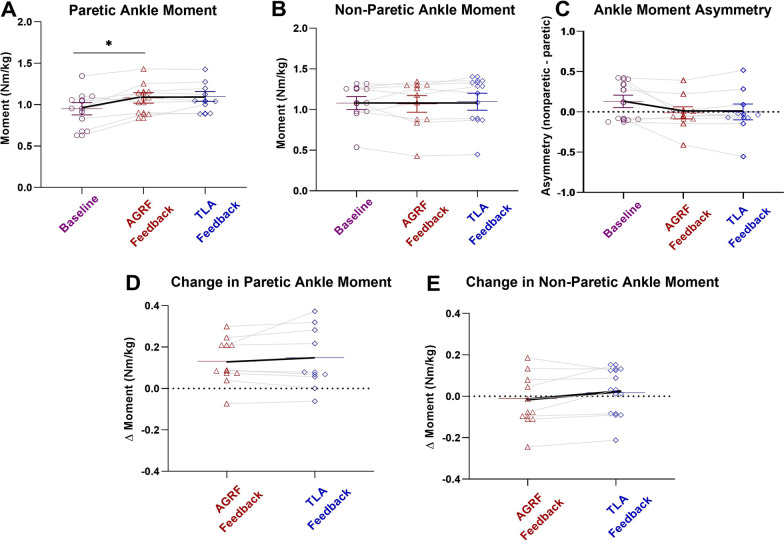
Peak ankle moment (average and standard errors) for the **A** paretic limb, **B** non-paretic limb, and **C** ankle moment asymmetry during baseline (no-biofeedback), AGRF biofeedback, and TLA biofeedback gait trials, as well as **D** change in paretic ankle moment and **E** change in non-paretic ankle moment with biofeedback. The one-way repeated measures ANOVA demonstrated a significant main effect of biofeedback on paretic limb peak ankle moment (p = 0.003). The symbol * indicates pairwise comparisons that showed significant differences between conditions. AGRF biofeedback (p = 0.031) induced significant increase in paretic limb peak ankle moment compared to baseline, while TLA biofeedback had no significant effect on ankle moment compared to baseline

The one-way repeated measures ANOVA demonstrated a significant main effect of biofeedback on paretic limb peak ankle moment during late stance (p = 0.003, F = 8.697). Pairwise comparisons revealed a significant increase in peak ankle moment during late stance during AGRF biofeedback trial compared to baseline (p = 0.031) (Fig. 3A). No significant differences in peak ankle moment during late stance were observed between the baseline and TLA biofeedback trials (p = 0.059), and AGRF biofeedback and TLA biofeedback trials (p = 1.000).

The one-way repeated measures ANOVA did not show a significant main effect of biofeedback on non-paretic limb peak ankle moment during late stance (p = 0.747, F = 0.297) (Fig. 3B).

### Interlimb peak ankle moment asymmetry

The one-way repeated measures ANOVA demonstrated a significant main effect of biofeedback on interlimb peak ankle moment asymmetry (p = 0.038, F = 4.058) (Fig. 3C). Bonferroni-corrected pairwise comparisons revealed no significant differences in interlimb peak ankle moment asymmetry between baseline and AGRF biofeedback trials (p = 0.130), baseline and TLA biofeedback trials (p = 0.302), and AGRF biofeedback and TLA biofeedback trials (p = 1.000).

### Paretic and non-paretic step length

The one-way repeated measures ANOVA did not show a significant main effect of biofeedback on paretic step length (p = 0.298, F = 1.308).

The one-way repeated measures ANOVA demonstrated a significant main effect of biofeedback on non-paretic step length (p < 0.001, F = 17.134). Pairwise comparisons revealed a significant increase in non-paretic step length during the AGRF biofeedback trial compared to baseline (p = 0.006). Additionally, pairwise comparisons revealed a significant increase in non-paretic step length during the TLA biofeedback trial compared to baseline (p = 0.003). A significant increase in non-paretic step length was observed during the TLA biofeedback trial compared to the AGRF biofeedback trial (p = 0.003).

### Inter-limb asymmetry in step length (paretic step length ratio)

The one-way repeated measures ANOVA demonstrated a significant main effect of biofeedback on step length ratio (p = 0.002, F = 9.305). Bonferroni-corrected pairwise comparisons revealed no significant difference in step length ratio during the AGRF biofeedback trial compared to baseline (p = 0.065). A significant change in paretic step length ratio was observed during the TLA biofeedback trial compared to baseline (p = 0.024). There were no significant differences in paretic step length ratio during the AGRF biofeedback trial compared to TLA biofeedback trial (p = 0.825).

## Discussion

We examined the immediate effects of TLA and AGRF biofeedback on kinetic and kinematic gait variables of post-stroke individuals. When compared to a no-feedback control trial, in response to both ARGF and TLA real-time audiovisual biofeedback, participants increased paretic limb AGRF and paretic TLA, while decreasing interlimb asymmetry of both AGRF and TLA. Furthermore, TLA biofeedback induced larger paretic limb TLA increases compared to AGRF biofeedback. In conjunction with feedback-induced increases in TLA, we observed increases in non-paretic leg step length for both types of gait feedback. Overall, participants were able to meet the target provided during gait biofeedback. AGRF biofeedback also significantly increased peak ankle moment. Taken together, our results suggest that real-time TLA biofeedback elicits immediate, targeted changes of the paretic limb post-stroke, like the results of a recent study on able-bodied individuals [26].

### Both AGRF and TLA biofeedback increased paretic AGRF and TLA

When TLA is targeted with feedback in people post-stroke, paretic leg AGRF increases concomitantly with TLA. Similarly, when AGRF is targeted with feedback, paretic leg TLA increases concomitantly with AGRF. This study is the first, to our knowledge, that directly shows concurrent improvements in post-stroke paretic TLA and propulsion with real-time TLA biofeedback. These parallel increases of propulsion and TLA are consistent with our hypothesis that AGRF and TLA are inter-related biomechanical parameters. Cross sectional studies have previously shown that individuals with greater propulsion also tend to have a larger TLA [19]. Here, concurrent intervention-induced modulation of AGRF and TLA in the same post-stroke cohort further supports the inter-relationships between these two variables. Hsiao et al. showed that stroke survivors who improved AGRF with gait training comprising fast treadmill walking and functional electrical stimulation also improve TLA. Furthermore, our results demonstrate that TLA and AGRF increase concomitantly with both types of biofeedback. This finding is promising because clinicians may be able to assess TLA more readily than AGRF subjectively (using observational gait analysis) [19] or objectively (using wearable sensors) [29]. Based on our results, modification of an individual’s TLA through targeted interventions and biofeedback warrants further exploration and would be predicted to be accompanied by an increase in propulsion. Increases in paretic propulsion and TLA will in turn be hypothesized to increase gait speed and reduce inter-limb asymmetries [8, 10, 17].

### Both AGRF and TLA feedback improved inter-limb asymmetries

Our study showed that, as seen in previous results with unilateral gait biofeedback among able-bodied individuals [26], both AGRF and TLA biofeedback are able to specifically target the paretic limb so that post-stroke inter-limb asymmetries are improved. Irrespective of the type of biofeedback, we demonstrated the immediate improvements in inter-limb asymmetries in propulsion and TLA with biofeedback. Furthermore, both means of biofeedback demonstrated limb-specific targeting of AGRF and TLA with no significant changes in the non-paretic limb. Paired with a trend of kinetic and kinematic normalization in the paretic limb, an overall normalization of inter-limb coordination of gait mechanics may be induced without a major change in the non-paretic limb. Thus, these short-term, immediate biofeedback-induced improvements in stroke gait demonstrate that post-stroke individuals have the neuromechanical capacity to improve their biomechanical deficits and restore inter-limb asymmetries.

As a caveat, TLA asymmetry worsened or reversed during TLA biofeedback for some stroke participants, such that the paretic leg demonstrated a larger TLA than the non-paretic leg. In people post-stroke, who typically have a shorter TLA on the paretic leg, this reversal of asymmetry such that the paretic leg now has a larger TLA may be deemed an undesired effect of TLA feedback. Perhaps this information, which can be gleaned using a short duration walking bout exposing the individual to biofeedback, could be utilized to personalize the target biofeedback variable. In other words, for those individuals who show reversal of TLA asymmetry with TLA feedback, perhaps AGRF feedback or a lower % TLA target would be a better biofeedback training strategy. Furthermore, a reversal of TLA asymmetry, while not ideal, does show that there is considerable reserve or capacity for paretic leg TLA to be increased post-stroke, and that normalization of TLA asymmetry may be achieved through biofeedback-based gait training interventions. Biofeedback-induced improvements of gait performance were recorded in short 60-s trials of AGRF and TLA biofeedback, further underscoring the immediacy of biofeedback-induced perturbation of gait patterns, and potential benefits of biofeedback as a post-stroke intervention.

### TLA biofeedback and AGRF biofeedback may improve post-stroke gait through different biomechanical mechanisms

Our comparison between the two modes of biofeedback showed that both TLA biofeedback and AGRF biofeedback increase paretic propulsion. However, TLA biofeedback induced larger increases in TLA than AGRF biofeedback. Somewhat surprisingly, significantly larger increases in TLA caused by TLA biofeedback versus AGRF biofeedback did not translate to larger feedback-induced increases in propulsion. Furthermore, compared to no-biofeedback, AGRF biofeedback effectively increased ankle moment despite participants receiving no specific instructions on increasing ankle moment or force generation as a strategy. In contrast, TLA biofeedback did not increase ankle moment. However, these results about TLA feedback not increasing ankle moment must be interpreted with some caution due to the preliminary nature of the study with a relatively small sample size. The larger TLA-induced increases in TLA may explain the ability of TLA biofeedback to induce similar increases in AGRF as AGRF biofeedback, despite TLA-biofeedback failing to increase ankle moment. Potentially, TLA biofeedback increased AGRF via modulation of overall limb positioning and less so via modulation of ankle-centered kinetics.

Future studies will require a systematic evaluation of the dose response relationship between TLA feedback and AGRF feedback targets. Such an approach may further enhance our understanding of the inter-relationships between these two variables. Most importantly, irrespective of the type of feedback, individuals post-stroke demonstrated the ability to modulate the targeted variables of the paretic limb without altering the non-paretic limb.

### Effect of TLA biofeedback and AGRF biofeedback on able-bodied participants versus post-stroke individuals

A comparison of our current results with our previous study on able-bodied participants [26] suggest that both able-bodied and post-stroke individuals were able to demonstrate immediate unilateral increases in AGRF in response to real-time biofeedback. Moreover, TLA biofeedback induced larger increases in targeted limb TLA for both able-bodied [26] and post-stroke individuals. Notably, unilateral TLA biofeedback increased bilateral TLA for able-bodied participants [26] while only preferentially increasing paretic TLA of post-stroke individuals. Able-bodied individuals naturally possess inter-limb symmetrical gait, and thus may have adapted to unilateral biofeedback through a modified gait pattern that consequently led to bilateral TLA modulation. Lastly, AGRF biofeedback but not TLA biofeedback increased ankle moment in both able-bodied [26] and post-stroke individuals, contributing to the evidence that TLA is influenced by overall limb position instead of ankle-centric strategies.

### Limitations and future directions

The limitations of our study included a small sample size, short duration of 60 s gait trials, and the use of a handrail during walking. The short walking bouts were related to the goal of the study being focused on evaluating immediate effects of biofeedback, and not short-term motor learning or retention. Another limitation was that due to the known biomechanical relationships between AGRF and TLA, and our previous findings that AGRF biofeedback induces concomitant increases in TLA, we had concerns that the introduction of TLA biofeedback first would elicit a learning strategy of exposing the individual to a specific biomechanical strategy to improve their AGRF. We, therefore, did not randomize the order of feedback trials in the current preliminary study, which is another methodological limitation. We posited that the introduction of AGRF biofeedback trial first, using the cue “push into the ground harder”, would incur lesser risk of eliciting specific biomechanical strategies in the subsequent TLA feedback gait trial. Future studies should investigate the appropriate dosage of biofeedback, long-term effects of biofeedback training, neurophysiologic correlates underlying the effect of biofeedback, and evaluate overground gait training with biofeedback. Additionally, future studies should explore the effect of gait biofeedback when variability in stroke severity or chronicity is accounted for. Lastly, longer exposure to gait biofeedback (e.g., multiple 6-min training bouts) will enable future investigations to evaluate retention of biofeedback-induced improvements in gait biomechanics, and potentially identify characteristics of responders versus non-responders to biofeedback.

## Conclusions

Our results show that TLA real-time biofeedback is a feasible and effective strategy to induce preferential and significant increases in paretic propulsion and TLA, while minimizing concomitant increases in non-paretic biomechanics of post-stroke individuals. These targeted biomechanical changes led to an improvement in inter-limb gait asymmetry. For post-stroke individuals, in contrast to AGRF biofeedback, TLA biofeedback induced an increase in paretic AGRF through modulation of overall limb positioning rather than ankle kinetics. By demonstrating the short-term effects of real-time TLA in comparison to AGRF biofeedback on gait biomechanics of post-stroke individuals, our results pave the way for future work exploring long-term retention and gait training with TLA biofeedback, strengthen our knowledge of the inter-relationships between TLA and AGRF, and underscore the potential for paretic TLA as a clinically modifiable post-stroke gait deficit.

## Data Availability

The datasets used and/or analysed during the current study are available from the corresponding author on reasonable request.

## Abbreviations

AGRF: Anterior-ground reaction forces
TLA: Trailing limb angle

## References

[1] Cruz TH, Lewek MD, Dhaher YY (2009). Biomechanical impairments and gait adaptations post-stroke: multi-factorial associations. J Biomech.

[2] Wonsetler EC, Bowden MG (2017). A systematic review of mechanisms of gait speed change post-stroke. Part 1: spatiotemporal parameters and asymmetry ratios. Top Stroke Rehabil.

[3] Weerdesteyn V, de Niet M, van Duijnhoven HJR, Geurts ACH (2008). Falls in individuals with stroke. J Rehabil Res Dev.

[4] Fritz S, Lusardi M (2009). White paper: “walking speed: the sixth vital sign”. J Geriatr Phys Ther.

[5] Perry J, Garrett M, Gronley JK, Mulroy SJ (1995). Classification of walking handicap in the stroke population. Stroke.

[6] Mahtani GB, Kinnaird CR, Connolly M, Holleran CL, Hennessy PW, Woodward J (2017). Altered sagittal- and frontal-plane kinematics following high-intensity stepping training versus conventional interventions in subacute stroke. Phys Ther.

[7] Kwakkel G, Lannin NA, Borschmann K, English C, Ali M, Churilov L (2017). Standardized measurement of sensorimotor recovery in stroke trials: consensus-based core recommendations from the stroke recovery and rehabilitation roundtable. Int J Stroke.

[8] Bowden MG, Behrman AL, Neptune RR, Gregory CM, Kautz SA (2013). Locomotor rehabilitation of individuals with chronic stroke: difference between responders and nonresponders. Arch Phys Med Rehabil.

[9] Bowden MG, Balasubramanian CK, Neptune RR, Kautz SA (2006). Anterior–posterior ground reaction forces as a measure of paretic leg contribution in hemiparetic walking. Stroke.

[10] Hsiao H, Awad LN, Palmer JA, Higginson JS, Binder-Macleod SA (2016). Contribution of paretic and nonparetic limb peak propulsive forces to changes in walking speed in individuals poststroke. Neurorehabil Neural Repair.

[11] Tyrell CM, Roos MA, Rudolph KS, Reisman DS (2011). Influence of systematic increases in treadmill walking speed on gait kinematics after stroke. Phys Ther.

[12] Lewek MD, Raiti C, Doty A (2018). The presence of a paretic propulsion reserve during gait in individuals following stroke. Neurorehabil Neural Repair.

[13] Franz JR, Kram R (2014). Advanced age and the mechanics of uphill walking: a joint-level, inverse dynamic analysis. Gait Posture.

[14] Conway KA, Bissette RG, Franz JR (2018). The functional utilization of propulsive capacity during human walking. J Appl Biomech.

[15] Hsiao H, Knarr BA, Higginson JS, Binder-Macleod SA (2015). The relative contribution of ankle moment and trailing limb angle to propulsive force during gait. Hum Mov Sci.

[16] Sylvester AD, Lautzenheiser SG, Kramer PA (2021). Muscle forces and the demands of human walking. Biol Open.

[17] Awad LN, Binder-Macleod SA, Pohlig RT, Reisman DS (2015). Paretic propulsion and trailing limb angle are key determinants of long-distance walking function after stroke. Neurorehabil Neural Repair.

[18] Hsiao H, Knarr BA, Higginson JS, Binder-Macleod SA (2015). Mechanisms to increase propulsive force for individuals poststroke. J Neuroeng Rehabil.

[19] Lewek MD, Sawicki GS (2019). Trailing limb angle is a surrogate for propulsive limb forces during walking post-stroke. Clin Biomech.

[20] Genthe K, Schenck C, Eicholtz S, Zajac-Cox L, Wolf S, Kesar TM (2018). Effects of real-time gait biofeedback on paretic propulsion and gait biomechanics in individuals post-stroke. Top Stroke Rehabil.

[21] Aiello E, Gates DH, Patritti BL, Cairns KD, Meister M, Clancy EA, et al. Visual EMG biofeedback to improve ankle function in hemiparetic gait. In: 2005 IEEE engineering in medicine and biology 27th annual conference. IEEE; 2005. p. 7703–6. 10.1109/IEMBS.2005.1616297 17282066

[22] Intiso D, Santilli V, Grasso MG, Rossi R, Caruso I (1994). Rehabilitation of walking with electromyographic biofeedback in foot-drop after stroke. Stroke.

[23] Moreland JD, Thomson MA, Fuoco AR (1998). Electromyographic biofeedback to improve lower extremity function after stroke: a meta-analysis. Arch Phys Med Rehabil.

[24] Miyazaki T, Kawada M, Nakai Y, Kiyama R, Yone K (2019). Validity of measurement for trailing limb angle and propulsion force during gait using a magnetic inertial measurement unit. Biomed Res Int.

[25] Saleh M, Murdoch G (1985). In defence of gait analysis. Observation and measurement in gait assessment. J Bone Joint Surg Br vol.

[26] Liu J, Santucci V, Eicholtz S, Kesar TM (2021). Comparison of the effects of real-time propulsive force versus limb angle gait biofeedback on gait biomechanics. Gait Posture.

[27] Kesar TM, Binder-Macleod SA, Hicks GE, Reisman DS (2011). Minimal detectable change for gait variables collected during treadmill walking in individuals post-stroke. Gait Posture.

[28] Liu J, Kim HB, Wolf SL, Kesar TM (2020). Comparison of the immediate effects of audio, visual, or audiovisual gait biofeedback on propulsive force generation in able-bodied and post-stroke individuals. Appl Psychophysiol Biofeedback.

[29] Porciuncula F, Roto AV, Kumar D, Davis I, Roy S, Walsh CJ (2018). Wearable movement sensors for rehabilitation: a focused review of technological and clinical advances. PM&R.

